# Predictive value of repeated cerebrospinal fluid parameters in the outcomes of bacterial meningitis in infants <90 days of age

**DOI:** 10.1371/journal.pone.0238056

**Published:** 2020-08-28

**Authors:** Joseph Y. Ting, Ashley Roberts, Sarah Khan, Ari Bitnun, Michael Hawkes, Michelle Barton, Jennifer Bowes, Jason Brophy, Lynda Ouchenir, Christian Renaud, Andrée-Anne Boisvert, Jane McDonald, Joan L. Robinson

**Affiliations:** 1 Department of Pediatrics, University of British Columbia, Vancouver, British Columbia, Canada; 2 Department of Pediatrics, McMaster University, Hamilton, Ontario, Canada; 3 Hospital for Sick Children & University of Toronto, Toronto, Ontario, Canada; 4 Department of Pediatrics, University of Alberta, Edmonton, Alberta, Canada; 5 Department of Pediatrics, Western University, London, Ontario, Canada; 6 Department of Pediatrics, University of Ottawa, Ottawa, Ontario, Canada; 7 Centre Hospitalier Universitaire Sainte-Justine, Université de Montréal, Montréal, Quebec, Canada; 8 Montréal Children's Hospital, McGill University, Montréal, Quebec, Canada; University of Oklahoma Health Sciences Center, UNITED STATES

## Abstract

**Background:**

There are variations in recommendations from different guidelines regarding the indications for repeat lumbar puncture (LP) in young infants with the diagnosis of bacterial meningitis.

**Objective:**

To evaluate the frequency of repeat LPs and the characteristics of cerebrospinal fluid (CSF) parameters in repeated sampling and their predictive values for adverse outcomes in a national cohort.

**Methods:**

This cohort study included infants born January 1, 2013 through December 31, 2014, who had proven or suspected bacterial meningitis within the first 90 days of life at seven paediatric tertiary care hospitals across Canada, and who underwent a repeat LP at the discretion of the treating physicians.

**Results:**

Forty-nine of 111 infants (44%) underwent repeat LP at a median of 5 (IQR: 3, 13) days after the LP that led to the diagnosis of bacterial meningitis. Those who had meningitis caused by gram negative bacilli were more likely to have repeat LP than those with gram positive bacteria (77% versus 57%; p = 0.012). White blood cell (WBC) count on the second spinal tap yielded an area under the curve of 0.88 for predicting sequelae of meningitis at discharge from the hospital, with a cut-off value of 366 × 10^6^/L, providing a sensitivity of 91% and specificity of 88%.

**Conclusion:**

In this multi-centre retrospective cohort study, infants with gram negative meningitis were more likely to have repeated LP. A high WBC on the second CSF sample was predictive of adverse outcome at the time of discharge from the hospital.

## Introduction

Neonatal bacterial meningitis is a life-threatening disease with high rates of mortality and morbidity and leads to substantial long-term neuro-disability [1–3]. Early recognition of meningitis and prompt treatment with appropriate antimicrobials is essential in optimizing outcome [4].

Repeat lumbar puncture (LP) during the course of treatment of bacterial meningitis and prolongation of antimicrobials for persistently abnormal cerebrospinal fluid (CSF) parameters have been advocated in young infants [5, 6]. However, there are variations in the guidelines to specify the indications for repeat LP or interpretation of results [7–10]. A 2001 survey in the United Kingdom revealed that only 18% of physicians routinely repeated CSFs in neonatal meningitis [11]. The objective of this study was to evaluate the frequency of repeat LP, the characteristics of CSF parameters in repeated sampling and their predictive values for adverse outcomes in a recent Canadian cohort of infants up to 90 days of age.

## Methods

The primary report on epidemiology, management, and outcomes of bacterial meningitis in this cohort of infants was previously published [5].

This cohort study included infants born January 1, 2013 through December 31, 2014 with onset of proven or suspected bacterial meningitis within the first 90 days of life at seven paediatric tertiary care hospitals across Canada. Infants underwent repeat LP at the discretion of the treating physicians. Infants with meningitis caused by fungus or virus, related to neurosurgical procedures or CSF shunts were excluded.

The study results are reported following the Strengthening the Reporting of Observational Studies in Epidemiology guidelines (https://www.equator-network.org/reporting-guidelines/strobe/). Ethics approval was obtained from the seven participating hospital Research Ethics Boards for this retrospective study. The cases were identified according to the International Statistical Classification of Diseases and Related Health Problems, Tenth Revision, and Canada Diagnostic Codes for Target Discharge [5].

Proven bacterial meningitis was defined as the detection of bacteria from CSF by culture or molecular techniques during life or at autopsy. Suspected meningitis was defined as the detection of bacteria known to cause central nervous system infection from blood or another normally sterile site (including urine) along with either sterile CSF pleocytosis or head imaging consistent with bacterial meningitis. CSF pleocytosis was defined as > 30 × 10^6^/L white blood cells (WBC) and (1) < 100 × 10^6^/L red blood cells (RBC), or (2) WBC:RBC ratio > 1:100 [12, 13]. The exclusion criteria were: (1) growth of a common skin contaminant (including coagulase-negative staphylococci) from a single CSF culture, (2) full recovery despite ≤ 4 days of intravenous antimicrobial therapy, or (3) fungal isolate [5].

The data were collected by Research Electronic Data Capture. The data included demographic characteristics, microbiological results (including drug susceptibilities), CSF results, head imaging results (any infarcts, abscesses, intracranial calcifications, hydrocephalus, intraventricular haemorrhage, parenchymal haemorrhage, etc.), duration of antimicrobial therapy, complications during hospital stay and sequelae noted at discharge, including ongoing seizures, use of anticonvulsants despite no ongoing seizures, documented hearing loss, abnormal findings on ophthalmological examination, increased tone, decreased tone, monoplegia/hemiplegia/quadriplegia/or other sequelae.

SPSS 26.0 (IBM Corp., Armonk, New York, USA) was used to perform the statistical analyses. Descriptive statistics were used to characterize the patient demographics and CSF parameters along with the outcomes. Kolmogorov-Smirnov test or Shapiro-Wilk test was applied to test for normality. The Mann-Whitney U test or Kruskal-Wallis test was performed as appropriate. The χ2 test or Fisher exact test was performed to compare the proportions of the categorical variables. A receiver operating characteristic curve was constructed to determine the threshold of CSF WBC for predicting adverse outcomes. A p value < 0.05 was considered as statistically significant.

## Results

**Fig 1:**
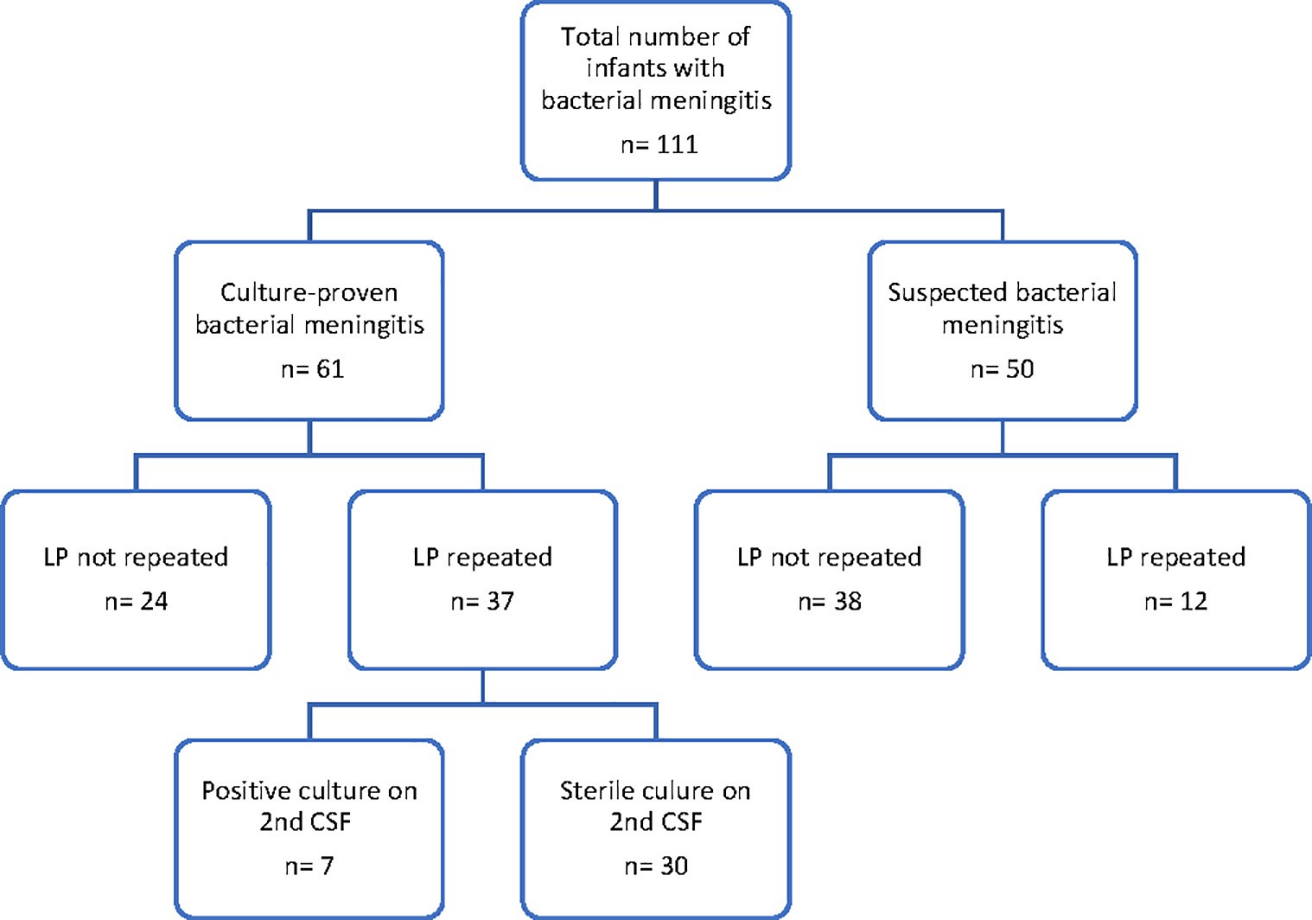
Study subjects. *n*: *number; LP*: *lumbar puncture; CSF*: *cerebrospinal fluid*.

After excluding infants with pre-existing CSF shunt (N = 1) and post-endoscopic ventriculostomy (N = 1), 111 infants with proven or suspected bacterial meningitis were included, with 44 (40%) born preterm [Fig 1]. Thirty-seven of 61 infants with proven meningitis (61%) and 12 of 50 infants with suspected meningitis (24%) underwent repeated LP. Variations were found in the practice of obtaining second CSF samples at different centres, ranging from 22% to 82% of infants (p = 0.005) across the seven sites. Nineteen infants (17%) had ≥ 3 CSFs obtained.

### Comparison of characteristics of infants with proven or suspected bacterial meningitis with and without repeat *LP* (n = 111)

There were no differences in gestational age (GA), birth weight (BW), sex and days of life (DOL) of diagnosis of meningitis with and without repeat LP [Table 1]. Those who had meningitis caused by gram negative bacilli were more likely to have repeat LP than those with gram positive bacteria (77% versus 57%; p = 0.012).

**Table 1:** Characteristics of infants with proven or suspected bacterial meningitis with and without repeat CSF sampling.

	Without repeated CSF sampling (n = 62)	With repeated CSF sampling (n = 49)	p-value
GA at birth (weeks), median, IQR	37 [32, 37]	37 [29, 37]	.633
Male, n (%)	34 (55%)	26 (53%)	.852
Birth weight (grams), median, IQR	2945 [1593, 3393]	2900 [1369, 3303]	.588
Day of life of diagnosis of meningitis, median, IQR	20 [11, 34]	17 [8, 30]	.620
Initial CSF findings, WBC x 10^6^/L, median, IQR	364 [95, 1774]	1084 [288, 4565]	.055
Organisms yielded initially from CSF			.012
Gram positive bacteria	15/35 (43%)	20/35 (57%)	
Gram negative bacilli	5/22 (23%)	17/22 (77%)	
Gram negative cocci	4/4 (100%)	0/4 (0%)	

n: number; GA: gestational age; CSF: cerebrospinal fluid; WBC: white blood cell count; IQR: interquartile range

The pathogens isolated from infants with proven bacterial meningitis (n = 61) are summarised in Table 2. Sixteen of 24 (66%) of infants with group B streptococcal (GBS) meningitis had repeated LP versus 10/14 (71%) of infants with *Escherichia coli* meningitis. The proportion of *E*. *coli* strains susceptible to ampicillin (10% vs. 0%, p = 0.714), gentamicin (80% vs. 50%, p = 0.311) and cefotaxime (80% vs. 50%, p = 0.311) showed no significant differences among infants with and without repeat CSF obtained. All infants with meningitis due to gram negative bacilli other than *E*. *coli* or *Klebsiella* sp. underwent repeat LP [Table 2].

**Table 2:** Microbiology of pathogen among infants with initial culture-proven CSF cultures.

Name of pathogen	No repeat CSF sampling	Repeat CSF sampling
**Total**	24	37
**Gram positive bacteria**		
*Streptococcus agalactiae*	8/24 (33.3%)	16/24 (66.7%)
*Streptococcus pneumoniae*	0/1 (0%)	1/1 (100%)
*Enterococcus spp*.	1/1 (100%)	0/1 (0%)
*Streptococcus bovis* group	2/4 (50%)	2/4 (50%)
*Coagulase-negative Staphylococcus spp*.	1/1 (100%)	0/1 (0%)
*Staphylococcus aureus*	1/1 (100%)	0/1 (0%)
*Listeria monocytogenes*	2/3 (66.7%)	1/3 (33.3%)
**Gram negative bacilli**		
*Escherichia coli*	4/14 (28.6%)	10/14 (71.4%)
*Klebsiella pneumoniae*	0/2 (0%)	2/2 (100%)
*Klebsiella oxytoca*	1/1 (100%)	0/1 (0%)
*Enterobacter cloacae*	0/2 (0%)	2/2 (100%)
*Enterobacter sakasakii*	0/1 (0%)	1/1 (100%)
*Cronobacter spp*.	0/1 (0%)	1/1 (100%)
*Serratia marcescens*	0/1 (0%)	1/1 (100%)
**Gram negative cocci**		
*Neisseria meningitidis*	4/4 (100%)	0/4 (0%)

### Infants with repeat LP (n = 49)

There were no differences of baseline demographic characteristics and microbiology between infants who underwent repeat LP with suspected (n = 12) and proven meningitis (n = 37), though the latter group had earlier repeat of CSF samples and higher median WBC in the second CSF samples [Table 3].

**Table 3:** Characteristics of 49 neonates with proven or suspected bacterial meningitis and repeat CSF sampling.

	Suspected meningitis (n = 12)	Proven meningitis (n = 37)	p-value
GA at birth (weeks), median, [IQR]	37 [29, 37]	37 [30, 37]	.685
Male	7/12 (58%)	19/37 (51%)	.674
Birth weight (grams), median, [IQR]	2730 [909, 3425]	2900 [1468, 3286]	.617
Day of life of onset of meningitis median, [IQR]	10 [2, 26]	18 [11, 37]	.056
No. of days between 1^st^ and 2^nd^ CSF samples Median, [IQR]	18 [7, 24]	4 [3, 9]	<0.001
Initial CSF WBC x 10^6^/L, median, [IQR]	536 [238, 2129]	1375 [363, 4761]	.202
Second CSF WBC x 10^6^/L, median, [IQR]	38 [7, 187]	262 [115, 1057]	.005
Pathogens			
Gram positive bacteria	3/12 (25%)	21/37 (54%)	.080
	*Bacillus spp (1)*	*GBS (16)*	
	*Group B Streptococcus (2)*	*Streptococcus pneumoniae (1)*	
		*Listeria monocytogenes (1)*	
		*Streptococcus bovis (2)*	
Gram negative bacilli	9/12 (75.0%)	17/37 (46%)	
	*Escherichia coli (5)*	*Escherichia coli (10)*	
	*Klebsiella spp*. *(1)*	*Klebsiella spp*. *(2)*	
	*Serratia spp*. *(1)*	*Serratia spp*. *(1)*	
	*Enterobacter spp*. *(1)*	*Enterobacter spp*. *(3)*	
	*Haemophilus*. *influenzae (1)*	*Cronobacter spp*. *(1)*	

GA: gestational age; GBS: group B streptococcus; IQR: interquartile range; CSF: cerebrospinal fluid; WBC: white blood cell

The median time chosen for repeat LP was 5 days (IQR: 3, 13) after the initial LP, with no difference between infants with proven or suspected bacterial meningitis caused by gram positive versus gram negative pathogens (4 versus 7 days, p = 0.178). The second sample was obtained < 7 days (28/49; 57%), 7 to 14 days (11/49; 22%) and > 14 days (10/49; 20%) after the initial LP. There was no significant variation of practice of CSF samplings beyond 14 days after the initial diagnosis among different centres (p = 0.444).

### Characteristics of infants with persistent positive bacterial culture on repeat LP (n = 7)

Seven of 37 (19%) infants with initial positive CSF culture had persistent positive bacterial cultures on the second CSF sample. This included infants with meningitis caused by *E*. *coli* (n = 2), GBS (n = 3), and one each of *Klebsiella pneumoniae* and *Enterobacter cloacae*. The CSF cell count in neither the initial nor second sample differentiated infants with persistent positive from sterile CSF cultures at repeat sampling (Table 4).

**Table 4:** CSF parameters in the first and second sampling of 37 infants with proven bacterial meningitis.

		**Persistent positive culture on second CSF (n = 7)	**Sterile culture on second CSF (n = 30)	p-value*
WBC (10^6^/L)	Initial CSF^@^	1600 [1087, 1749]	1150 [426, 4664]	.763
	Second CSF^@^	351 [241, 929]	227 [120, 962]	.418

WBC: white blood cell count; CSF: cerebrospinal fluid

^@^ Expressed as Median [Interquartile Ranges]

* Mann-Whitney U tests

** Limited to the infants with culture proven meningitis in their first CSF samples

### Time to CSF sterilization in infants with repeat LP (n = 37)

**Fig 2:**
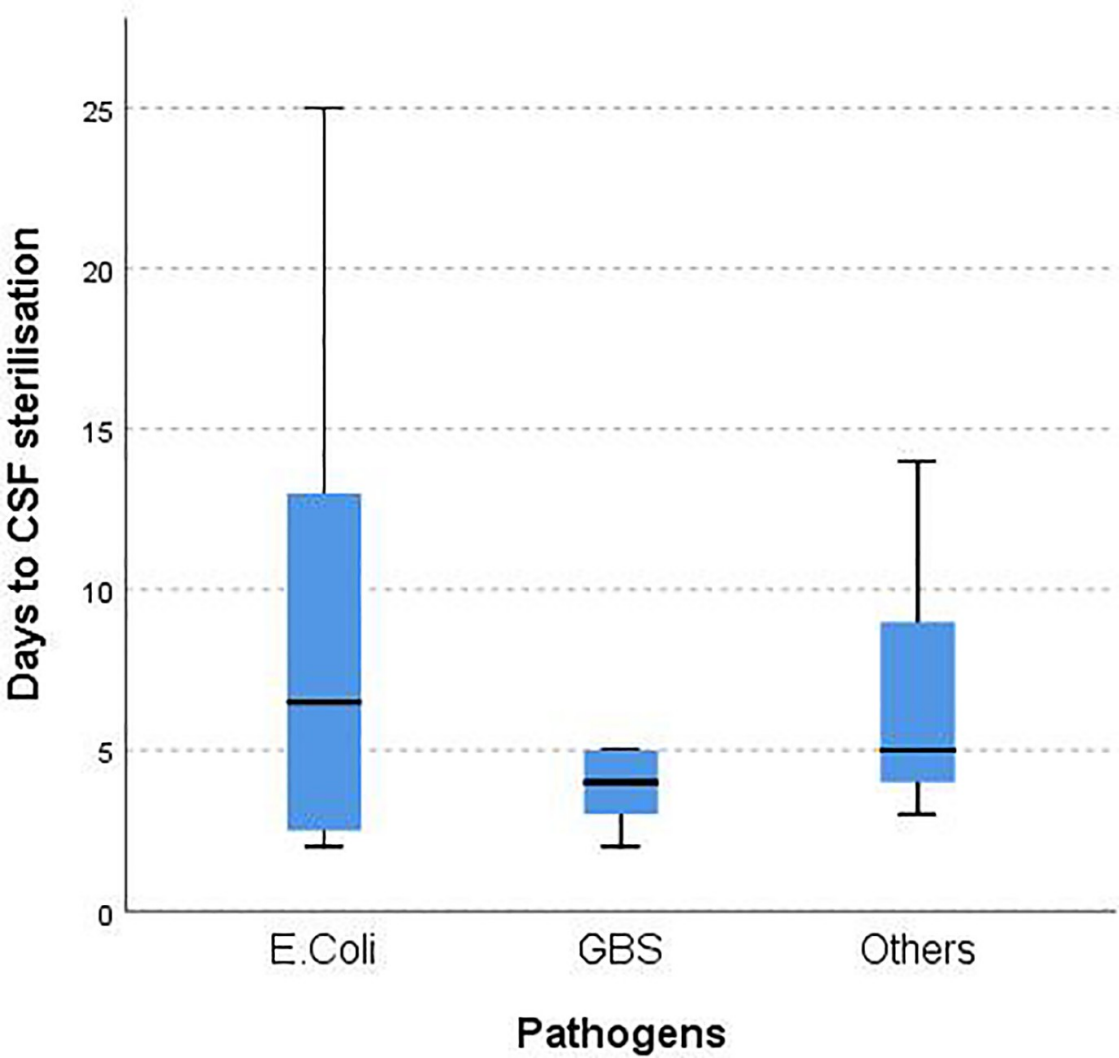
Time to CSF sterilization in infants with repeat LP. *CSF*: *cerebrospinal fluid*. By Kruskal-Wallis test; p = 0.656.

Thirty out of 37 infants with bacterial growth on the initial CSF had sterile CSF on their second LP [Fig 1]. Median time to document CSF sterilization among infants with meningitis caused by *E*.*coli* (n = 8), GBS (n = 13) and other bacterial pathogens (n = 9) were 7, 4, and 5 days, respectively [Fig 2], with no significant differences between the three groups (p = 0.656).

### Outcomes in infants with repeat LP—sequelae apparent at discharge from the hospital (n = 49)

All infants survived to hospital discharge, so diagnosis was never made at autopsy. Twelve of 49 infants (24%) had at least one sequelae noted at the time of discharge, namely ongoing seizures (n = 10), hearing loss (n = 1), abnormal tone (n = 2) and/or others (n = 4). There was no significant difference in the proportion with sequelae between term (6/29, 23%) and preterm infants (6/29 [21%] vs. 6/20, [30%], p = 0.456).

**Fig 3:**
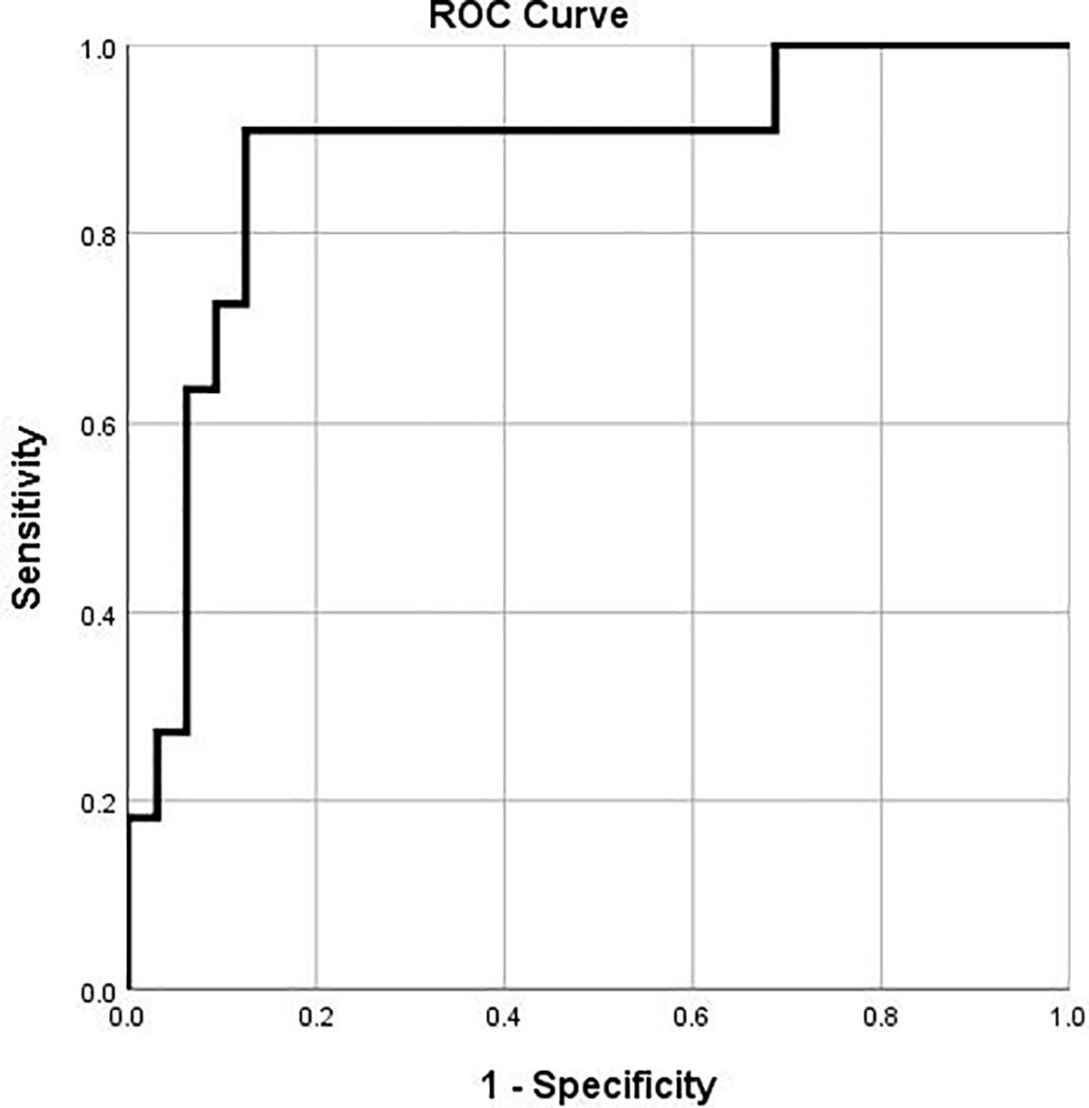
CSF WBC from the 2^nd^ LP to predict complications at discharge from hospital. A cut-off value of 366 × 10^6^/L, provided a sensitivity of 91% and specificity of 88%. *CSF*: *cerebrospinal fluid; WBC*: *white blood cell count; LP*: *lumbar puncture*.

WBC in the first CSF sample or the magnitude of decrease in WBC in from the first to second CSF samples were not found to be predictive of sequelae at hospital discharge [Table 5]. However, WBC on the second LP yielded an area under the curve (AUC) of 0.881 (95% CI, 0.753−1.000; p<0.001) for predicting sequelae at discharge from hospital, with a cut-off value of 366 × 10^6^/L, providing a sensitivity of 91% and specificity of 88% [Fig 3].

**Table 5:** Predictive value of CSF white cell counts for adverse outcomes at hospital discharge.

Parameter	AUC	95% CI	p-value*
1^st^ CSF sample WBC	.666	.477-.854	.118
2^nd^ CSF sample WBC	.881	.753–1.000	<0.001
Difference between 1^st^ & 2^nd^ CSF samples^@^	.598	.419-.777	.381

CSF: cerebrospinal fluid; WBC: white blood cell count

Analysis performed using receiver operating curves (ROC); AUC: area under the curve

^@^ (2nd CSF WBC– 1st CSF WBC)/1^st^ CSF WBC

* Null hypothesis: AUC = 0.5

In this cohort, persistent positive culture on the second CSF samples was not predictive of sequelae at hospital discharge (22% [2/7] versus 23% [7/30] with sterile cultures (2/7 [22%] vs. 7/30 [23%], p = 0.556).

## Discussion

In our cohort, almost half of infants with suspected or proven bacterial meningitis had CSF sampled at least twice during their treatment course with repeat sampling being more common with gram-negative than with gram-positive meningitis. Approximately 60% of repeat sampling was performed within 7 days of the initial sampling. There was no difference in the median days to document CSF sterilization among infants with meningitis caused by different pathogens. Our ROC analysis revealed that elevated CSF WBC of 366 x 10^6^/L or above at the second LP was predictive of sequelae at the time of hospital discharge.

Some experts recommend that all infants with bacterial meningitis undergo repeat LP 48 hours after initiation of therapy [7]. For infants in whom the initial CSF is obtained so early in the course that CSF findings of bacterial meningitis are not definitive, the repeat LP 24 to 48 hours later may provide resolution, as there is considerable overlap between CSF parameters in neonates with and without bacterial meningitis [14]. Repeat LP is also useful where antimicrobial resistance to the usual therapies is proven or suspected which might explain why repeat sampling was done in the current study for all infants with gram negatives other than *E*. *coli*. [15]. In the era of rising numbers of multi-drug resistant organisms in hospitals and even in the community, we cannot deny the potential benefits of repeat LP in infants with meningitis caused by multi-drug resistant organisms (e.g. 3^rd^-generation cephalosporin-resistant Enterobacteriaceae, β-lactam-resistant *Streptococcus pneumoniae)* to monitor the clinical response and document clearance of the organism [5, 16]. In fact, in the management guideline published by the Infectious Disease Society of North America, repeated CSF analysis is recommended for any patient who has not responded clinically after 48 hours of appropriate antimicrobial therapy [9].

However, the utility of repeat LP with meningitis due to pathogens that are not multidrug-resistant is less clear. In adult bacterial meningitis, repeat LP is not commonly performed, although it might be useful in selected cases to confirm the diagnosis, to exclude relapse or persistent infection, or for therapeutic purposes in communicating hydrocephalus [17]. Durack et al. reported that wide ranges of glucose and protein levels and cell counts at the end of treatment were compatible with cure in a series of 165 adults, and concluded that post-treatment lumbar puncture was of no value as a test of cure in bacterial meningitis [18]. Schaad UB et al. reported that CSF parameters at the end of therapy did not appear to be predictive of recrudescence or relapse of meningitis in children, comparing 8 children who were not cured by initial therapy to 22 controls. [19]

Some experts recommend performing repeat LP routinely in all neonates when meningitis is caused by gram-negative bacilli at 48 hours, whereas others suggest repeating LP only if clinical improvement is not evident after 24 to 72 hours of antibiotics [6, 8, 9, 20]. In our cohort, 44% (49/111) of infants underwent repeat LP. This rate was comparable to that reported in a large population cohort of infants from neonatal intensive care units (Pediatrix Medical Group, 55%) [7], but higher than that in the survey of the usual practice of physicians from north-west England in cases of neonatal meningitis (18%) [11]. The practice variation in obtaining repeat CSF among the participating centres in this cohort (22 to 82%) highlighted the lack of best practice guidance on repeating LP among infants with meningitis. It seems likely that early repeat sampling was done to identify infants with delayed CSF sterilization while late sampling was done to look for persistently abnormal CSF parameters, both of which might lead to prolonging therapy. However, there are no guidelines on interpretation of CSF parameters during therapy. Only 10 of 111 infants had CSF obtained more than 14 days after the initial CSF, suggesting that it is no longer common practice to obtain end-of-therapy CSF. We are not aware of any studies looking at the predictive value of end-of-therapy CSF parameters in young infants.

We demonstrated that the WBC on the second CSF sample but not the first CSF sample was predictive of adverse outcomes at the time of discharge from the hospital. Greenberg et al. reported the presence of a positive culture in a repeat CSF was associated with increased mortality [7]. Studies by Tan et al. revealed that high CSF protein after two weeks of antimicrobial therapy was associated with poor outcome at 0 to 3 months of age, though the Glasgow Outcome Scale used for outcome assessment has limited validity in young children [21–23].

To the best of our knowledge, this is the first study evaluating the usefulness of repeat LP in predicting adverse neonatal outcomes other than mortality among infants with bacterial meningitis. The major limitation of the study is that long-term outcome data were inconsistently available and not collected in a standardized manner. The total number of subjects was relatively small. Details of brain imaging were not recorded systematically to allow meaningful analysis. Adverse outcomes could have been related to other factors rather than to bacterial meningitis (e.g. hemodynamic instability during hospitalisation). The timing of repeat LP was not consistent. The decision to repeat the LP was at the discretion of the treating physician and the rationale was typically not documented. The database did not capture the initial clinical presentation nor any failed repeat LP attempts. The relatively small number of subjects did not allow us to carry out further multivariate analysis to look at the contributing factors towards adverse sequelae. Having said that, our study indicated that repeat LP may provide insights on prognostication. Future prospective studies with large sample sizes should analyze the decision making process regarding repeat LP, the results and interpretation of CSF parameters and the relationship between CSF parameters and long-term outcomes in samples obtained with uniform timing (possibly 3 days after effective antibiotics were started).”

## Conclusions

In this multi-centre retrospective cohort study, we found that infants with gram negative meningitis were more likely to have repeated LP. A high WBC on the second CSF sample was predictive of adverse outcomes at the time of discharge from the hospital.
